# The relationship between dietary nutrient intake and adolescent depression based on the dietary inflammation index: a case-control study

**DOI:** 10.1186/s12888-026-08425-y

**Published:** 2026-08-03

**Authors:** Junxiang Cheng, Ning Ma, Qia liu, Guofang Yang, Chenjing Ma, Hong Han, Na liu, Zhifen Liu, Shifan Han

**Affiliations:** 1 Department of Psychiatry, The First Hospital of Shanxi Medical University, Taiyuan, 030001 China; 2 Department of Neurosurgery, Shanxi Cardiovascular Hospital, Taiyuan, 030024 China; 3 School of Nursing, Shanxi Medical University, Taiyuan, 030001 China; 4 Department of Magnetic Resonance Imaging, Shanxi Bethune Hospital, Taiyuan, 030032 China; 5 Department of Plastic Surgery, The First Hospital of Shanxi Medical University, Taiyuan, 030001 China; 6 Shanxi Key Laboratory of Artificial Intelligence Assisted Diagnosis and Treatment for Mental Disorder, Taiyuan, 030001 China

**Keywords:** Major depressive disorder (MDD), Adolescent, Energy-adjusted Dietary Inflammatory Index (E-DII)

## Abstract

**Background and purpose:**

The global increase in adolescent Major Depressive Disorder (MDD) has profound implications for physical and mental development, contributing to a significant societal burden. Emerging evidence suggests that low-grade chronic inflammation, commonly observed in adolescents with MDD, is associated with the severity of the disorder. This study explores the relationship among dietary inflammation, dietary nutrients and adolescent depression, utilizing data from the Energy-adjusted Dietary Inflammatory Index (E-DII) in a population from northern China.

**Methods:**

A case-control study was conducted between March 2022 and October 2023, involving 172 treatment-naïve adolescents aged 10–23 years diagnosed with MDD and 172 age- and sex-matched healthy controls. Although the calculated minimum sample size was 174 per group, two matched pairs were excluded during data cleaning because extreme dietary reporting could have biased nutrient intake estimation. The E-DII was derived using data from a validated semi-quantitative Chinese food frequency questionnaire (FFQ). Logistic regression was employed to evaluate the association between E-DII and the risk of adolescent MDD, while restricted cubic spline (RCS) functions assessed the dose-response relationships between specific dietary nutrient intakes and MDD.

**Results:**

A positive association was identified between the E-DII and the risk of adolescent MDD (OR = 3.24; 95% CI: 2.32–4.51; *P* < 0.001), which persisted after adjusting for potential confounders (OR = 3.23; 95% CI: 2.22–4.69; *P* < 0.001). Although an increase in E-DII score correlated with higher HAMD scores, intra-group differences were not statistically significant (*P* = 0.159). Several dietary nutrients, including Vitamin B12, dietary fiber, zinc, polyunsaturated fatty acids (PUFA), folate, and anthocyanins, were negatively associated with the risk of MDD in adolescents, whereas carbohydrates, selenium, and alcohol intake were positively associated with MDD risk (all *P* < 0.05). RCS analysis showed nonlinear dose–response relationships for PUFA, anthocyanins, vitamin B12, and alcohol intake, whereas selenium, zinc, folate, carbohydrates, and dietary fiber showed linear associations with MDD risk.

**Conclusion:**

This study underscores a positive correlation between the E-DII and both the risk and severity of adolescent depression, emphasizing the critical role of dietary factors in the prevention and management of adolescent depression. From a clinical and public health perspective, early dietary assessment and promotion of anti-inflammatory dietary patterns may provide a feasible adjunctive strategy for reducing depression risk in adolescents.

**Supplementary Information:**

The online version contains supplementary material available at https://doi.org/10.1186/s12888-026-08425-y.

## Introduction

Major depressive disorder (MDD) is a prevalent mental health condition characterized by frequent recurrence, significant impairment in daily functioning, and a high risk of suicide, affecting both physical and mental health [1]. The onset of MDD often occurs in early adolescence, with risk increasing linearly throughout this period and the global prevalence among this age group rose by 13% from 2001 to 2020 [2]. “China National Mental Health Development Report (2021–2022),” revealed that 14.8% of surveyed youth face moderate to severe MDD risk, making depression a significant threat to the physical and mental well-being of Chinese adolescents [3]. Severe cases can lead to suicidal and self-harming thoughts, and even death. Despite advances in mental health interventions, the burden of depressive disability-adjusted life-years (DALYs) continues to grow [4]. Therefore, exploring effective strategies for preventing and treating MDD is crucial to improving adolescents’ quality of life.

The etiology and pathogenesis of adolescent depression are not fully understood. Since Maes (1995) introduced the inflammation hypothesis of MDD, research has increasingly shown that MDD often involves an immune response marked by elevated levels of pro-inflammatory cytokines, such as tumor necrosis factor-alpha (TNF-α), interleukin (IL), and interferon-gamma [5]. Accumulating evidence suggests that low-grade chronic inflammation is involved in the pathophysiology of adolescent depression. Elevated circulating inflammatory markers, including C-reactive protein and pro-inflammatory cytokines, have been reported in adolescents with depressive symptoms, and inflammatory activity appears to correlate with symptom severity [6]. Persistent neuroinflammation can lead to glucocorticoid receptor desensitization and glucocorticoid resistance, disrupting the hypothalamic-pituitary-adrenal (HPA) axis response and diminishing the anti-inflammatory effects of glucocorticoids. Additionally, pro-inflammatory cytokines can increase the activity of indoleamine 2,3-dioxygenase (IDO), reducing the bioavailability of tryptophan, a precursor to serotonin. Neuroinflammatory responses can cause neuronal excitotoxicity and hinder the production of brain-derived neurotrophic factor (BDNF), resulting in the degeneration of neural circuits involved in emotion regulation [7].

Systemic inflammation related to MDD has various causes, with diet playing a critical role as a lifelong exposure factor. On average, individuals consume approximately 60 tons of food over their lifetime [8]. In recent decades, global dietary habits have shifted from high-fiber, nutrient-rich diets to those high in saturated fats, refined grains, and sugars. Chinese dietary patterns have also changed significantly, with trends showing excessive energy intake, increased consumption of high-calorie foods, preference for energy-dense options like meat snacks and beverages [9]. Although China currently has the lowest percentage of DALYs attributable to neurological and psychiatric disorders among individuals over 75, younger generations are experiencing rapidly increasing DALYs that surpass those in the United States and other countries [1].

The Dietary Inflammatory Index (DII) is an assessment tool developed by researchers at the University of South Carolina to evaluate the potential inflammatory effects of an individual’s diet [10]. Chronic low-grade inflammation has been increasingly recognized as an important biological mechanism in the pathophysiology of MDD, and that the DII is a validated tool to quantify the inflammatory potential of diet. It categorizes diets on a continuum from most anti-inflammatory to most pro-inflammatory based on their impact on specific inflammation markers. The initial DII version, based on data from 1950 to 2007, assigned scores to foods and ingredients based on their effects on markers such as IL-1β, IL-6, TNF-α, CRP, IL-4, and IL-10. In 2014, the DII was refined to incorporate overall dietary patterns and a comprehensive database representing diverse global diets, enhancing its applicability worldwide. The DII can be derived from a 24-hour dietary recall or food record data, evaluating the potential of various foods to influence inflammation through their effects on pro-inflammatory and anti-inflammatory markers. Given the emerging evidence linking dietary inflammation to depression, but the limited data in adolescent populations among northern China, this study aimed to investigate the association between the energy-adjusted Dietary Inflammatory Index (E-DII), specific dietary nutrient intake, and the risk and severity of major depressive disorder among adolescents in northern China.

## Methods

### Study population

This study conducted from March 2022 to October 2023 at the First Hospital of Shanxi Medical University. It focuses on untreated patients experiencing depressive episodes that meet the ICD-10 F32 diagnostic criteria. A 1:1 control group of healthy individuals has been included. Previous research has shown an odds ratio (OR) of 3.98 regarding the potential link between dietary inflammation and MDD risk. The control group had a population factor exposure ratio of 0.33 [11]. With α = 0.05, β = 0.10, and Phi = 0.20, and using a CPT matching principle, the sample size was estimated using PASS 11.0 (NCSS, USA). The analysis indicated that a minimum sample size of 174 cases is required for each group. Two patients were excluded because implausible dietary reporting was identified during data cleaning, which could have biased nutrient intake estimation. The corresponding matched controls were therefore removed, leaving 172 participants in each group.

Inclusion and Exclusion Criteria: for the case group:

Inclusion criteria: (1) Meeting ICD-10 F.32 criteria for a depressive episode; (2) Aged 10 to 23 years which consistent with the expanded contemporary definition of adolescence proposed by Sawyer et al., which extends adolescence from 10 to 24 years of age [12]; (3) First consultation with no prior treatment; (4) Effective communication without language barriers; Exclusion criteria: (1) Medical conditions affecting eating habits (e.g., hypertension, diabetes, hyperlipidemia, cardiovascular disease, gout, digestive diseases, fatty liver, or malignant tumors); (2) Coexisting mental disorders; (3) Pregnancy; (4) Participants who had used anti‑inflammatory, vitamin supplements, mineral supplements, herbal nutritional products, or other nutritional support within the previous three months were excluded to reduce potential bias in dietary intake assessment.

For the control group: inclusion criteria: Healthy individuals aged 10 to 23 years. Exclusion criteria: (1) Previous or current mental illness diagnosis; (2) Medical conditions affecting eating habits; (3) Pregnancy; (4) Participants who had used anti‑inflammatory, vitamin supplements, mineral supplements, herbal nutritional products, or other nutritional support within the previous three months were excluded to reduce potential bias in dietary intake assessment.

Family history of diabetes and hypertension was recorded as indicators of familial metabolic background because these conditions may be associated with long-term dietary patterns and systemic inflammatory status.

The study protocol was approved by the Ethics Committee of the First Hospital of Shanxi Medical University. Written informed consent was obtained from all participants and guardians. (Ethic No: K062).

### Data collection

Trained researchers conducted face-to-face interviews to gather clinical information and dietary data. Clinical data included age, gender, education level, smoking and drinking history, medical and family history, height, and weight. BMI was calculated by dividing weight (kg) by height (m^2^).

Depressive symptoms were evaluated using the 24-item Hamilton Depression Rating Scale (HAMD-24). All interviews were conducted by trained psychiatrists who received unified training before the study to ensure scoring consistency. HAMD-24 has demonstrated good internal consistency (Cronbach’s alpha = 0.90) and reliability (0.88) [13]. Dietary intake was assessed using a validated semi-quantitative Chinese FFQ previously applied in Chinese populations, which included 149 food items representing major dietary components [14]. During the interview, portion sizes were estimated using standardized food photographs and household measuring utensils to improve accuracy.

The Energy-Adjusted Dietary Inflammation Index (E-DII) was calculated based on Shivappa N et al.‘s [10] method, standardizing DII values to 1000 kilocalories to account for differences in total energy consumption. The FFQ collected data on 28 food parameters used in E-DII calculation, including, energy, carbohydrates, protein, fat, cholesterol, dietary fiber, vitamins (A, B6, B12, C, D, E), thiamine, riboflavin, niacin, folate, β-carotene, magnesium, iron, zinc, selenium, saturated fatty acids (SFAs), monounsaturated fatty acids (MUFAs), polyunsaturated fatty acids (PUFAs), n-6 and n-3 PUFAs, anthocyanins, and soybean isoflavones.

### Data analysis

The distribution of continuous variables was assessed using the Kolmogorov-Smirnov test. Variables with normal distributions were expressed as mean ± standard deviation and compared using the independent-samples Student’s t-test, whereas non-normally distributed variables were expressed as median (interquartile range) and analyzed using the Mann-Whitney U test.

Logistic regression models were used to estimate the association between dietary factors and adolescent depression. Multivariable models were adjusted for age, sex, education level, body mass index, alcohol consumption, family history of psychiatric disorders, family history of hypertension, and family history of diabetes. These covariates were selected based on previous evidence showing potential associations with both dietary exposure and depression. Statistical analysis was performed using SPSS 23.0 software, with a two-tailed P-value < 0.05 indicating significance. To elucidate the dose-response relationship between dietary nutrient intake and adolescent with MDD, a restricted cubic spline (RCS) function was utilized, with all analyses conducted using the ‘rms’, ‘ggplot2’, and ‘foreign’ packages in R 4.2.3. A relationship was considered linear when the overall association was statistically significant (*P* < 0.05) and the test for non-linearity was not significant (*P* for non-linearity > 0.05). A significant P value for non-linearity (*P* < 0.05) was interpreted as evidence of a non-linear association.

## Results

### General information of the research subjects

The study included 344 participants, with 172 in the case group and 172 in the control group. The mean age was 17.20 ± 2.84 years in the case group and 18.47 ± 1.30 years in the control group. No significant differences were found between the groups in age, education level, smoking status, alcohol consumption, and past medical history. In the case group, 36 participants (20.9%) had a positive family history of mental illness, significantly higher than the 10 (5.81%) in the control group (*P* = 0.006). Additionally, the BMI was significantly higher in the case group compared to the control group (24.25 ± 1.98 vs. 22.55 ± 1.93 kg/m²; *P* < 0.001). Table 1 provides detailed sociodemographic characteristics.

**Table 1:** Baseline characteristics of the study participants

Variables	Mean ± SD or *N* (%)		*P*- value
	Cases (*N* = 172)	Controls (*N* = 172)	
Age	17.20 ± 2.84	18.47 ± 1.30	0.13
Gender			1.00
Female	106 (61.6%)	106 (61.6%)	
Male	66 (38.4%)	66 (38.4%)	
Education			0.864
primary school	46 (26.7%)	49 (28.5%)	
Middle school	116 (67.4)	109 (63.4%)	
College	10 (5.8%)	14 (8.1%)	
Alcohol			0.615
Never	126 (73.3%)	118 (68.6%)	
Yes	46 (26.7%)	54 (31.4%)	
Psychiatric family history			**0.006**
Yes	36 (20.9%)	10 (5.8%)	
No	136 (79.1%)	162 (94.2%)	
Family history of diabetes			0.063
Yes	48 (27.9%)	26 (15.1%)	
No	124 (72.1%)	146 (84.9%)	
Family history of high blood pressure			0.054
Yes	36 (20.9%)	16 (9.3%)	
No	136 (79.1%)	156 (90.7%)	
BMI (kg/$\:{\mathrm{m}}^{2})$	24.25 ± 1.98	22.55 ± 1.93	**< 0.001**

BMI, body mass index; Data were analyzed by chi-squared test, independent Student’s t-tests or Fisher’s exact test

### Relationship between daily dietary nutrient intake, E-DII, and the risk of MDD in adolescents

The case group had a significantly higher daily total energy intake than the control group (3429.00 ± 946.88 vs. 2933.43 ± 819.65 kcal; *P* < 0.001). They also had higher daily intakes of protein, carbohydrates, selenium, soy isoflavones, and alcohol (all *P* < 0.05). Conversely, the case group consumed lower amounts of dietary fiber, magnesium, iron, zinc, PUFA, n-6 PUFAs, folate, vitamin C, β-carotene/carotenoids, anthocyanins, vitamin B12, and vitamin D (all *P* < 0.05). No significant differences were observed in the intake of fat, cholesterol, vitamin A, thiamine, riboflavin, niacin, vitamin E, saturated fatty acids, MUFAs, n-3 PUFAs, and vitamin B6 (all *P* > 0.05) (Table 2).

**Table 2:** Comparison of daily dietary nutrient intake between the case and control groups

Variables	Cases (*n* = 172) Mean ± SD/median (IQR)	Controls (*n* = 172) Mean ± SD/median (IQR)	T/Z	*P* - value^a^
Energy (kcal)	3429.00 ± 946.88	2933.42 ± 819.65	-3.67	**< 0.001**
Proteins (g)	108.21 ± 40.09	89.81 ± 29.11	-3.44	**0.001**
Total fats (g)	111.26 ± 49.35	106.48 ± 36.86	-0.72	0.473
Carbohydrates (g)	510.46 ± 194.15	412.40 ± 147.9	-3.73	**< 0.001**
Dietary fiber (g)	16.85 ± 7.37	24.88 ± 12.68	5.08	**< 0.001**
Cholesterol (mg)	634.96 ± 410.22	643.21 ± 308.46	0.15	0.882
Vitamin A (µg RE)	443.41 (220.24, 772.75)	575.18 (339.93, 869.67)	-0.15	0.31
Thiamine (mg)	1.11 ± 0.48	1.18 ± 0.44	0.99	0.323
Riboflavin (mg)	0.89 (0.68, 1.13)	0.85 (0.69, 1.03)	-1.00	0.320
Niacin (mg)	18.76 ± 9.38	20.21 ± 9.34	1.02	0.31
Vitamin C (mg)	445.53 ± 347.36	732.16 ± 499.70	5.33	**< 0.001**
Vitamin E (mg)	17.16 ± 8.18	15.72 ± 7.83	-1.18	0.24
Mg (mg)	471.81 ± 166.97	647.51 ± 302.95	4.71	**< 0.001**
Fe (mg)	33.22 ± 11.94	41.11 ± 19.06	3.26	**0.001**
Zn (mg)	15.34 ± 5.08	17.58 ± 6.81	2.45	**0.015**
Se (µg)	89.28 ± 35.83	61.54 ± 20.52	-6.23	**< 0.001**
SFA (g)	33.14 ± 17.71	29.52 ± 11.26	-1.60	0.112
MUFA (g)	50.39 ± 25.63	46.99 ± 18.50	-1.00	0.319
PUFA (g)	10.34 ± 5.58	18.09 ± 5.09	9.52	**< 0.001**
Folate (µg)	103.27 ± 43.34	164.88 ± 58.14	7.88	**< 0.001**
b-carotene (mg)	404.13 ± 397.65	574.90 ± 481.65	2.54	**0.012**
Anthocyanin (µg)	6.64 ± 4.03	10.37 ± 4.10	4.76	**< 0.001**
Isoflavone (mg)	0.03 (0.01, 6.30)	7.18 (3.26, 12.79)	-6.58	**< 0.001**
Alcohol (g)	11.32 ± 4.07	2.06 ± 0.86	-2.59	**0.011**
n-3PUFA (g)	5.52 ± 3.14	5.09 ± 2.77	-0.96	0.339
n-6PUFA (g)	34.54 ± 18.96	43.44 ± 18.77	3.09	**0.002**
VitaminB6 (mg)	0.78 ± 0.63	0.89 ± 0.55	1.25	0.211
VitaminB12 (µg)	0.063 ± 0.12	0.13 ± 0.04	5.38	**< 0.001**
Vitamin D (IU)	187.19 ± 77.76	387.58 ± 87.09	5.35	**< 0.001**
DII	0.85 ± 1.36	-1.06 ± 1.42	-9.01	**< 0.001**
E-DII	2.49 ± 1.21	-0.16 ± 1.63	-12.09	**< 0.001**

DII, dietary inflammatory index; E-DII, energy-adjusted dietary inflammatory index; ^a^ P-values were obtained from independent Student’s t-tests

Logistic regression analysis, adjusted for confounding factors, revealed negative associations between VB12, dietary fiber, zinc, PUFA, folate, and anthocyanin with the risk of MDD in adolescents: VB12 (OR = 0.87; 95% CI: 0.00-0.01; *P* < 0.001), dietary fiber (OR = 0.81; 95% CI: 0.75–0.87; *P* < 0.001), zinc (OR = 0.52; 95% CI: 0.29–0.93; *P* = 0.03), PUFA (OR = 0.52; 95% CI: 0.38–0.71; *P* < 0.001), folate (OR = 0.98; 95% CI: 0.96–0.99; *P* < 0.001), and anthocyanin (OR = 0.72; 95% CI: 0.61–0.85; *P* < 0.001). Positive associations were found with the intake of carbohydrates (OR = 1.01; 95% CI: 1.01–1.02; *P* < 0.001), selenium (OR = 1.29; 95% CI: 1.14–1.46; *P* < 0.001), and alcohol (OR = 1.21; 95% CI: 1.06–1.39; *P* = 0.01) (Table 3).

**Table 3:** Multivariable logistic regression analysis of dietary nutrient intake and the risk of MDD in adolescents

Variables	B	S.E.	Wald	df	Sig.	Exp (B)	95% CI. for EXP (B)	
							Lower	Upper
VitaminB12 (µg) ^a^	-10.03	2.896	12.00	1.00	0.00	0.17	0.12	0.20
Carbohydrates (g)^a^	0.01	0.003	13.37	1.00	0.00	1.01	1.01	1.02
Dietary fiber (g)^a^	-0.22	0.039	31.43	1.00	0.00	0.81	0.75	0.87
Zn (mg)^a^	-0.65	0.295	4.88	1.00	0.03	0.52	0.29	0.93
Se (µg) ^a^	0.26	0.062	16.68	1.00	0.00	1.29	1.14	1.46
PUFA (g)^a^	-0.66	0.158	17.37	1.00	0.00	0.52	0.38	0.71
Folate (µg) ^a^	-0.03	0.006	14.35	1.00	0.00	0.98	0.96	0.99
Anthocyanin (µg) ^a^	-0.33	0.086	14.34	1.00	0.00	0.72	0.61	0.85
Alcohol (g)^a^	0.19	0.070	7.41	1.00	0.01	1.21	1.06	1.39
Constant	3.26	0.748	19.02	1.00	0.00	26.10		

^a^ Fully adjusted for age, sex, education, smoking, past medical history, family history, and body mass index

The energy-adjusted E-DII value was significantly higher in the case group compared to the control group (2.49 ± 1.21 vs. -0.16 ± 1.63; *P* < 0.001). E-DII positively correlated with the risk of adolescent depression (OR = 3.24; 95% CI: 2.32–4.51; *P* < 0.001). After adjusting for confounding factors, this association persisted (OR = 3.23; 95% CI: 2.22–4.69; *P* < 0.001).

The E-DII scores ranged from − 1.55 to 5.52 in the case group and − 3.17 to 4.57 in the control group and SPSS was used for data conversion and binning. T1 (E-DII < 0.21) represented the lowest dietary inflammation potential, T2 (E-DII 0.21–2.35) indicated moderate potential, and T3 (E-DII > 2.35) exhibited the highest potential. The risk of MDD increased with higher E-DII scores (T2 OR = 16.10; 95% CI: 5.14–50.41; *P* < 0.001; T3 OR = 114.75; 95% CI: 30.6-430.32; *P* < 0.001). After adjustments, this association remained significant (T2 OR = 17.49; 95% CI: 4.89–62.49; *P* < 0.001; T3 OR = 100.9; 95% CI: 23.32-436.61; *P* < 0.001) (Table 4).

**Table 4:** Odds ratios and 95% confidence intervals for the association between E-DII and the risk of MDD in adolescents

	T1	T2	T3	E-DII continuous
E-DII Range	< 0.21	0.21–2.35	> 2.35	
Cases/Controls	8/108	62/52	102/12	172/172
OR (95%, CI)	1.0 (Ref)^*^	16.10 (5.14–50.41) ^*^	114.75 (30.6-430.32) ^*^	3.24 (2.32–4.51) ^*^
OR (95%, CI) ^a^	1.0 (Ref)^a*^	17.49 (4.89–62.49) ^a*^	100.9 (23.32-436.61) ^a*^	3.23 (2.22–4.69) ^a*^

^a^ Fully adjusted for age, sex, education, smoking, Alcohol, physical activity and body mass index; ^*^P<0.05; E-DII tertiles were defined according to the distribution of E-DII scores in the total study population

Energy intake was significantly different among T1, T2, and T3 groups in the control group (*P* = 0.001). Table 6 showed the dietary nutrient intake according to E-DII distribution. In the case group, increased intakes of protein, cholesterol, and selenium correlated with higher E-DII scores (all *P* < 0.05). Decreased intakes of vitamin E, magnesium, iron, zinc, folate, and soy isoflavones also correlated with higher E-DII scores (all *P* < 0.05). No significant differences were observed in fat, carbohydrates, dietary fiber, vitamin A, thiamine, riboflavin, niacin, pantothenic acid, vitamin C, vitamin B6, vitamin B12, vitamin D, n-3 PUFAs, n-6 PUFAs, MUFAs, saturated fatty acids, PUFAs, anthocyanins, and alcohol intake (all *P* > 0.05) (Table 5).

**Table 5:** Distribution of dietary nutrient intake across tertiles of E-DII (per 1,000 kcal)

Variables	Groups	T1(< 0.21)	T2(0.21–2.35)	T3(> 2.35)	*P*-value
Energy (kcal)	Cases	2476.93 ± 634.72	3388.05 ± 971.76	3619.2 ± 874.69	0.066
	Controls	2320.31 ± 519.67	2584.97 ± 604.50	3169.31 ± 849.00	**0.001**
Proteins (g)	Cases	63.03 ± 9.32	102.91 ± 37.31	122.76 ± 41.08	**0.005**
	Controls	65.62 ± 27.89	78.45 ± 22.55	97.97 ± 29.13	**0.001**
Total fats (g)	Cases	58.36 ± 21.43	119.87 ± 53.20	110.17 ± 46.31	0.06
	Controls	91.44 ± 32.61	99.53 ± 29.95	114.49 ± 37.56	**0.027**
Carbohydrates (g)	Cases	420.35 ± 81.51	484.39 ± 178.00	565.00 ± 219.13	0.12
	Controls	296.84 ± 104.85	352.73 ± 109.16	453.96 ± 152.81	**0.002**
Dietary fiber (g)	Cases	16.83 ± 7.92	19.13 ± 7.76	15.46 ± 6.88	0.09
	Controls	29.10 ± 12.31	19.48 ± 9.86	10.13 ± 5.86	**< 0.001**
Cholesterol (mg)	Cases	121.26 ± 61.85	618.78 ± 381.72	727.86 ± 434.78	**0.017**
	Controls	602.53 ± 302.58	628.23 ± 241.90	654.94 ± 340.47	0.888
Vitamin A (µg RE)	Cases	561.81 ± 48.34	905.57 ± 163.50	1591.25 ± 861.96	0.105
	Controls	1192.34 ± 928.69	467.38 ± 310.19	573.15 ± 223.85	0.127
Thiamine (mg)	Cases	0.75 ± 0.22	1.21 ± 0.51	1.07 ± 0.47	0.142
	Controls	1.30 ± 0.43	1.01 ± 0.34	0.77 ± 0.39	**< 0.001**
Riboflavin (mg)	Cases	0.89 ± 0.59	1.10 ± 0.54	0.92 ± 0.43	0.216
	Controls	1.02 ± 0.49	0.75 ± 0.27	0.65 ± 0.24	**0.013**
Niacin (mg)	Cases	12.79 ± 2.84	17.54 ± 8.97	21.53 ± 9.96	0.074
	Controls	22.45 ± 9.58	17.11 ± 7.44	13.52 ± 8.73	**0.009**
Vitamin C (mg)	Cases	439.01 ± 346.70	403.72 ± 568.28	305.90 ± 364.41	0.58
	Controls	785.62 ± 502.03	644.08 ± 420.30	632.70 ± 779.05	0.44
Vitamin E (mg)	Cases	22.16 ± 9.16	15.29 ± 5.81	14.26 ± 6.09	**< 0.001**
	Controls	18.10 ± 8.17	12.62 ± 5.29	7.71 ± 2.71	**< 0.001**
Mg (mg)	Cases	531.66 ± 166.47	493.36 ± 161.12	433.74 ± 159.55	**0.033**
	Controls	747.58 ± 297.90	525.11 ± 224.63	277.31 ± 129.63	**< 0.001**
Fe (mg)	Cases	38.15 ± 12.18	35.53 ± 8.14	30.03 ± 11.05	**0.009**
	Controls	47.25 ± 18.97	33.17 ± 14.41	20.30 ± 8.32	**< 0.001**
Zn (mg)	Cases	17.15 ± 5.27	14.40 ± 4.82	13.28 ± 3.50	**0.040**
	Controls	20.03 ± 6.44	14.24 ± 5.22	10.00 ± 4.43	**< 0.001**
Se (µg)	Cases	49.33 ± 12.22	84.81 ± 33.02	101.81 ± 37.38	**0.007**
	Controls	47.92 ± 17.78	52.65 ± 16.46	67.33 ± 20.59	0.362
SFA (g)	Cases	17.85 ± 11.33	33.06 ± 17.67	35.23 ± 17.88	0.182
	Controls	26.14 ± 6.47	27.56 ± 9.89	30.83 ± 12.19	**< 0.001**
MUFA (g)	Cases	21.55 ± 2.98	51.70 ± 23.17	51.86 ± 26.91	0.069
	Controls	38.44 ± 14.40	41.29 ± 14.06	51.73 ± 19.24	**0.007**
PUFA (g)	Cases	5.53 ± 1.56	10.57 ± 4.86	10.58 ± 6.06	0.212
	Controls	19.73 ± 4.83	16.66 ± 5.29	15.04 ± 4.15	**< 0.001**
Folate (µg)	Cases	121.92 ± 48.97	92.32 ± 31.95	92.79 ± 36.83	**0.01**
	Controls	184.23 ± 56.53	138.18 ± 41.88	106.42 ± 54.51	**< 0.001**
b-carotene (mg)	Cases	193.61 ± 114.17	517.09 ± 491.53	351.99 ± 329.91	0.105
	Controls	684.10 ± 535.41	414.28 ± 319.39	288.09 ± 182.83	**0.018**
Anthocyanin (µg)	Cases	8.30 ± 4.81	5.69 ± 3.74	2.27 ± 1.73	0.608
	Controls	12.75 ± 11.51	7.10 ± 7.04	3.27 ± 3.65	**0.015**
Isoflavone (mg)	Cases	17.37 ± 12.07	15.13 ± 6.92	4.06 ± 1.23	**< 0.001**
	Controls	13.24 ± 13.07	8.17 ± 6.95	1.33 ± 1.58	**0.018**
Alcohol (g)	Cases	5.13 ± 1.12	6.82 ± 6.71	7.77 ± 5.53	0.152
	Controls	0.93 ± 0.55	1.80 ± 0.86	2.28 ± 0.90	0.926
n-3PUFA (g)	Cases	5.84 ± 3.58	5.21 ± 2.41	3.83 ± 1.05	0.376
	Controls	5.69 ± 2.81	4.48 ± 4.11	3.98 ± 1.94	**0.029**
n-6PUFA (g)	Cases	28.49 ± 3.50	31.81 ± 122.75	36.68 ± 22.37	0.432
	Controls	46.19 ± 15.88	37.34 ± 15.00	45.13 ± 43.75	0.139
VitaminB6 (mg)	Cases	0.35 ± 0.08	0.92 ± 0.66	0.71 ± 0.62	0.149
	Controls	0.96 ± 0.59	0.76 ± 0.46	0.69 ± 0.58	0.223
VitaminB12 (µg)	Cases	0.08 ± 0.05	0.07 ± 0.04	0.06 ± 0.04	0.932
	Controls	0.13 ± 0.12	0.18 ± 0.14	0.05 ± 0.03	0.182
Vitamin D (IU)	Cases	252.54 ± 136.07	177.70 ± 130.99	192.50 ± 115.75	0.929
	Controls	408.75 ± 376.82	432.65 ± 357.42	171.66 ± 84.00	0.147

The One-way analysis of variance (ANOVA) was used for comparison of the variables between E-DII tristiles

T1: Cases (n = 8), Controls (n = 108); T2: Cases (n = 62), Controls (n = 52); T3: Cases (n = 102), Controls (n = 12)

### Relationship between dietary nutrient intake and the incidence of depression severity in adolescents

The total HAMD score classified depressive symptoms into three categories: mild (8–20 points), moderate (20–35 points), and severe (> 35 points). As shown in Tables 6 and 24 patients (14%) exhibited mild symptoms, 92 patients (53.4%) displayed moderate symptoms, and 56 patients (32.6%) presented with severe symptoms. Although the E-DII score tended to increase with higher HAMD scores, the differences among the groups were not statistically significant (*P* > 0.05).

Energy intake in the case group increased with severity: mild (2720.10 ± 763.29 kcal), moderate (3122.33 ± 815.29 kcal), and severe (4236.62 ± 646.05 kcal; all *P* < 0.05). Protein, fat, carbohydrates, riboflavin, selenium, MUFA, and n-6 PUFAs consumption also increased with severity (all *P* < 0.05). Conversely, dietary fiber, vitamin E, magnesium, iron, zinc, thiamine, PUFA, and n-3 PUFAs intake decreased with severity (all *P* < 0.05). No significant differences were observed in cholesterol, vitamin A, vitamin C, vitamin B6, vitamin B12, vitamin D, niacin, β-carotene/carotenoids, folate, anthocyanins, and soy isoflavones intake (all *P* > 0.05) (Table 6).

**Table 6:** Stratified analysis of dietary nutrient intake versus MDD severity

Variables	Mild (HAMD 8–20) *N* = 24 (14%)	Moderate (HAMD 20–35) *N* = 92 (53.4%)	Severe (HAMD > 35) *N* = 56 (32.6%)	*P*-value
Energy (kcal)	2720.10 ± 763.29	3122.33 ± 815.29	4236.62 ± 646.05	**< 0.001**
Proteins (g)	85.88 ± 34.56	100.60 ± 35.07	130.29 ± 41.21	**0.001**
Total fats (g)	92.70 ± 48.04	99.53 ± 45.65	138.48 ± 45.82	**0.001**
Carbohydrates (g)	391.07 ± 192.66	469.87 ± 150.66	628.33 ± 205.35	**< 0.001**
Dietary fiber (g)	19.73 ± 7.04	15.57 ± 6.98	15.03 ± 8.20	**0.039**
Cholesterol (mg)	630.45 ± 348.77	634.31 ± 413.74	637.98 ± 441.50	0.998
Vitamin A (µg RE)	406.14 (224.03, 803.91)	437.87 (241.35, 671.09)	443.40 (210.60, 750.84)	0.96
Thiamine (mg)	1.37 ± 0.51	0.99 ± 0.41	0.94 ± 0.43	**0.001**
Riboflavin (mg)	0.80 (0.50, 0.92)	0.83 (0.66, 1.07)	1.05 (0.89, 1.29)	0.006
Niacin (mg)	14.88 ± 6.48	18.58 ± 9.58	20.71 ± 9.81	0.195
Vitamin C (mg)	228.06 ± 163.60	332.59 ± 357.10	422.73 ± 605.72	0.43
Vitamin E (mg)	21.32 ± 8.33	15.49 ± 7.60	13.81 ± 6.51	**0.003**
Mg (mg)	568.00 ± 157.28	430.82 ± 147.73	404.53 ± 172.98	**0.001**
Fe (mg)	38.26 ± 11.67	31.36 ± 11.22	28.56 ± 12.26	**0.017**
Zn (mg)	18.20 ± 4.83	14.35 ± 4.58	12.49 ± 4.74	**< 0.001**
Se (µg)	68.51 ± 32.79	82.27 ± 30.94	109.73 ± 36.05	**< 0.001**
SFA (g)	26.28 ± 14.43	30.36 ± 16.83	40.64 ± 18.38	**0.017**
MUFA (g)	42.58 ± 24.69	43.65 ± 21.46	64.81 ± 27.05	**0.001**
PUFA (g)	12.58 ± 6.01	9.52 ± 4.95	8.23 ± 5.62	**0.025**
Folate (µg)	113.85 ± 47.29	96.99 ± 42.50	102.64 ± 34.47	0.27
b-carotene (mg)	265.21 ± 245.70	377.01 ± 368.43	508.24 ± 475.30	0.166
Anthocyanin (µg)	7.02 ± 5.13	7.07 ± 3.83	2.85 ± 2.26	0.440
Isoflavone (mg)	0.01 (0.01, 7.57)	0.08 (0.02, 6.33)	0.08 (0.01, 6.30)	0.347
Alcohol (g)	9.48 ± 5.73	7.82 ± 3.90	12.08 ± 3.66	0.861
n-3PUFA (g)	6.91 ± 3.94	4.67 ± 2.37	5.53 ± 2.72	**0.011**
n-6PUFA (g)	36.15 ± 10.01	29.65 ± 11.85	41.89 ± 27.52	**0.023**
VitaminB6 (mg)	0.49 ± 0.39	0.84 ± 0.66	0.78 ± 0.66	0.23
VitaminB12 (µg)	0.02 ± 0.02	0.04 ± 0.06	0.04 ± 0.07	0.32
Vitamin D (IU)	211.66 ± 140.46	190.13 ± 130.84	84.48 ± 46.36	0.317
E-DII	1.96 ± 1.74	2.46 ± 1.17	2.75 ± 0.93	0.159

The One-way analysis of variance (ANOVA) was used for comparison of the variables between mild, moderate and sever group among case group

The One-way analysis of variance (ANOVA) was used for comparison of the variables between mild, moderate and sever group among case group.

Ordered logistic regression analysis, controlling for confounding factors, showed significant associations between total energy intake and iron intake with depressive symptoms severity (both *P* < 0.05). Higher total energy intake was a risk factor (OR = 1.003; 95% CI: 0.001–0.004; *P =* 0.001), while increased iron intake was negatively associated with symptom severity (OR = 0.81; 95% CI: -0.385- -0.024; *P =* 0.026) (Table 7).

**Table 7:** Logistic regression analysis of dietary nutrient intake on MDD severity

Variables	B	S.E.	Wald	df	Sig.	Exp (B)	95% CI.for EXP (B)	
							Lower	Upper
[HAMD = 1] ^a^	0.917	4.101	0.050	1.00	0.823	2.50	-7.121	8.954
[HAMD = 2] ^a^	5.31	4.169	1.622	1.00	0.203	202.35	-2.861	13.482
Energy (kcal)^a^	0.003	0.001	11.118	1.00	0.001	1.003	0.001	0.004
Fe (mg)^a^	-0.205	0.092	4.938	1.00	0.026	0.81	0.68	0.98

^a^ Fully adjusted for age, sex, education, smoking, Alcohol, physical activity and body mass index; The proportional odds assumption was tested using the test of parallel lines and was satisfied (*P* > 0.05)

### Nonlinear relationship between dietary nutrient intake and the risk of MDD in adolescents

**Fig. 1:**
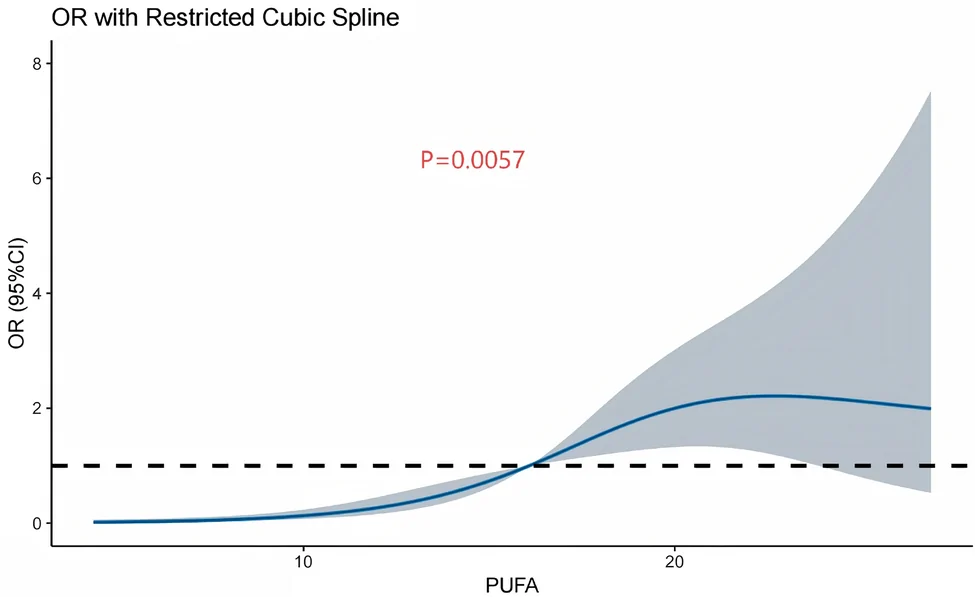
RCS analysis of PUFA intake and the risk of MDD in adolescents

**Fig. 2:**
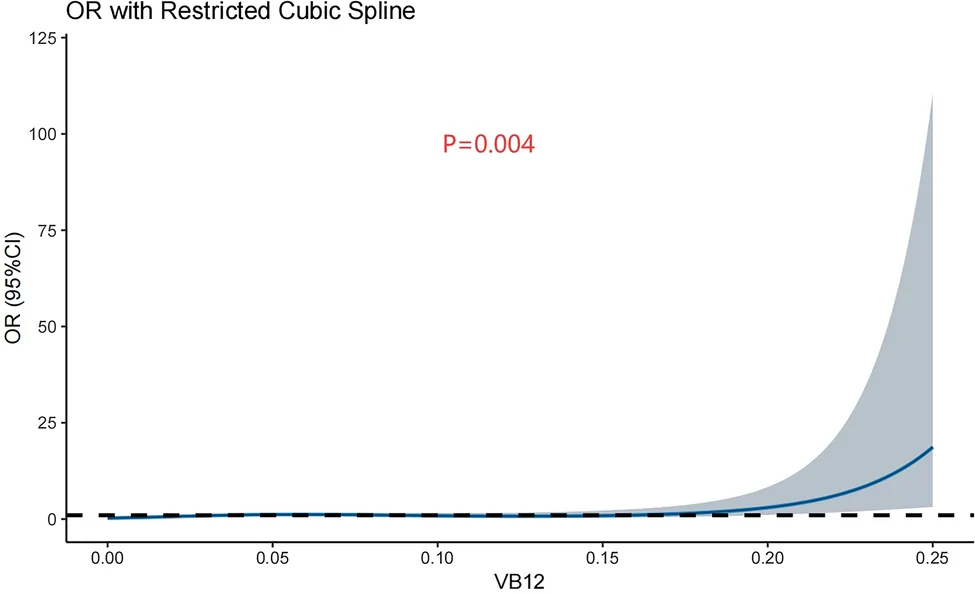
RCS analysis of vitamin B12 intake and the risk of MDD in adolescents

**Fig. 3:**
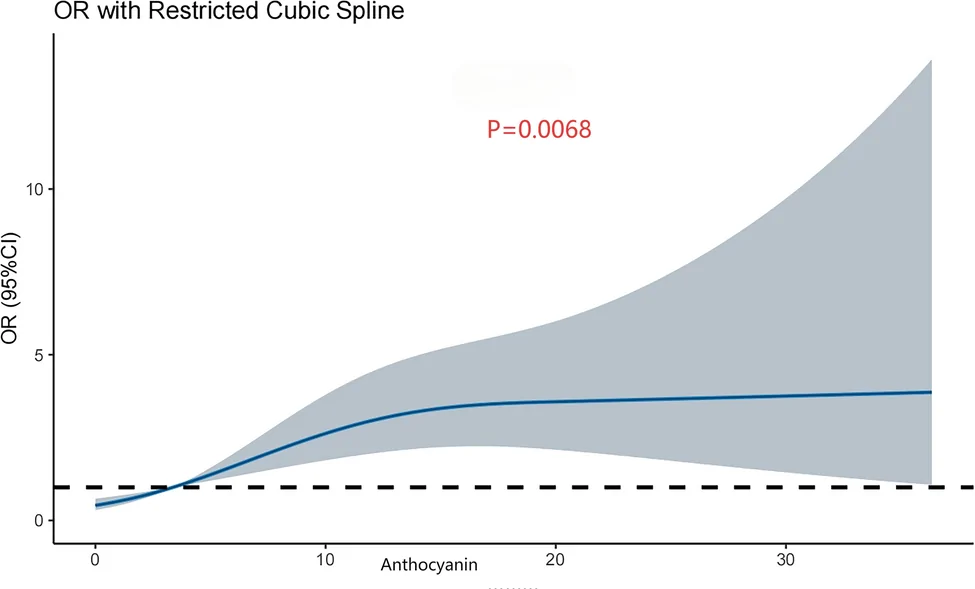
RCS analysis of anthocyanin intake and the risk of MDD in adolescents

**Fig. 4:**
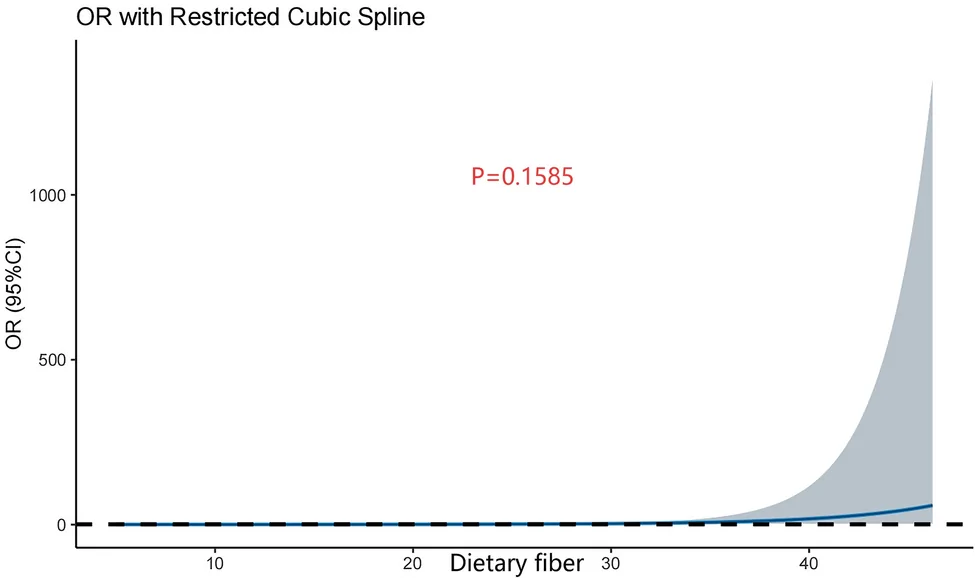
RCS analysis of dietary fiber intake and the risk of MDD in adolescents

**Fig. 5:**
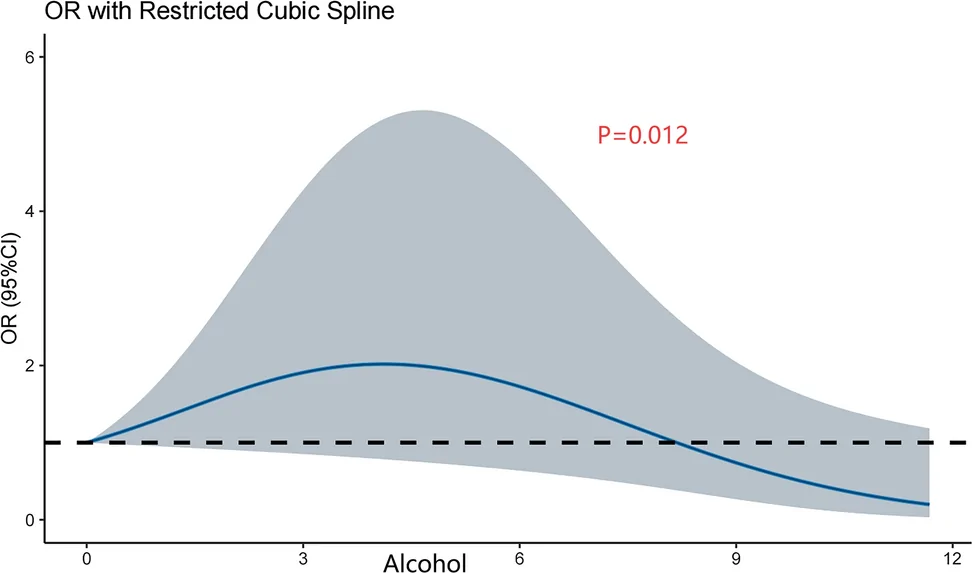
RCS analysis of alcohol intake and the risk of MDD in adolescents

**Fig. 6:**
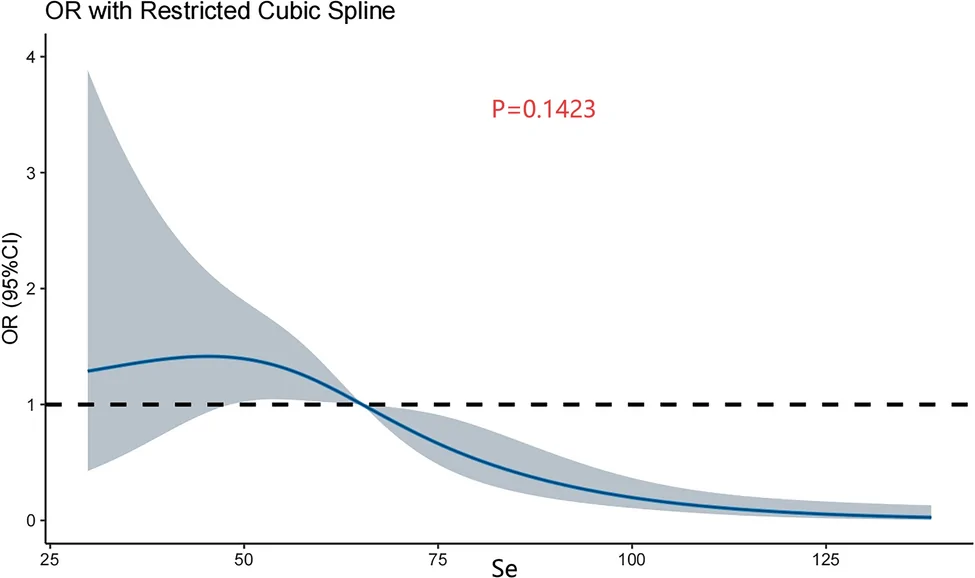
RCS analysis of selenium intake and the risk of MDD in adolescents

**Fig. 7:**
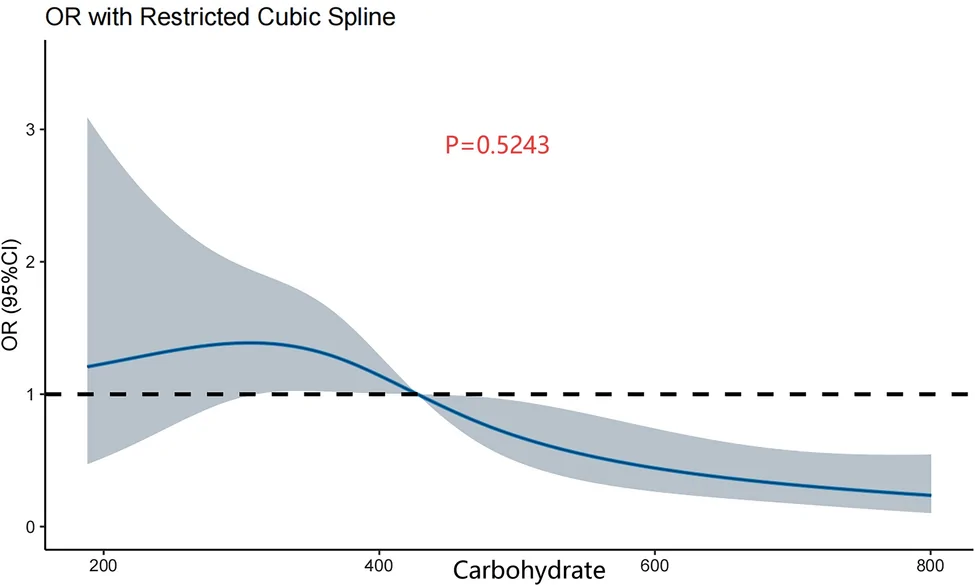
RCS analysis of carbohydrate intake and the risk of MDD in adolescents

**Fig. 8:**
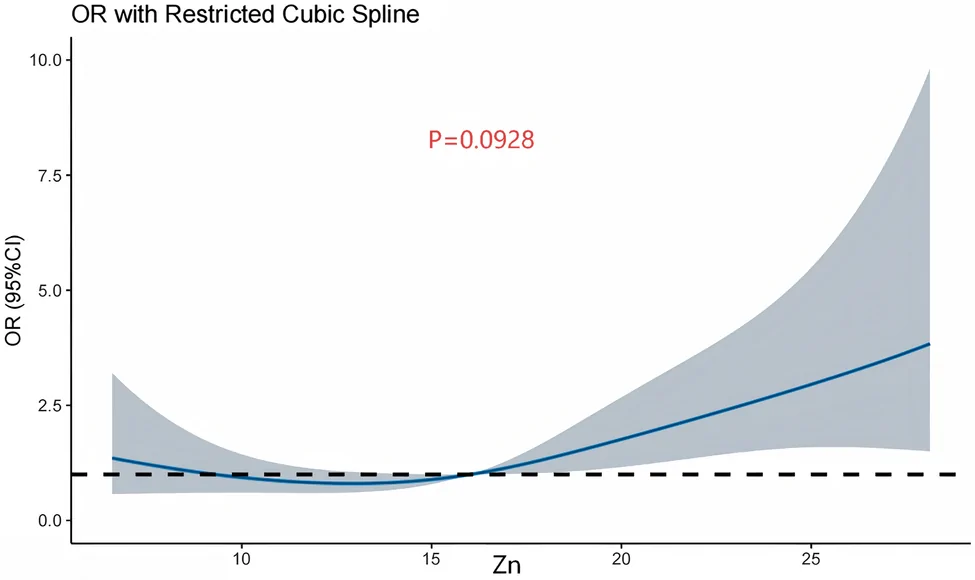
RCS analysis of zinc intake and the risk of MDD in adolescents

**Fig. 9:**
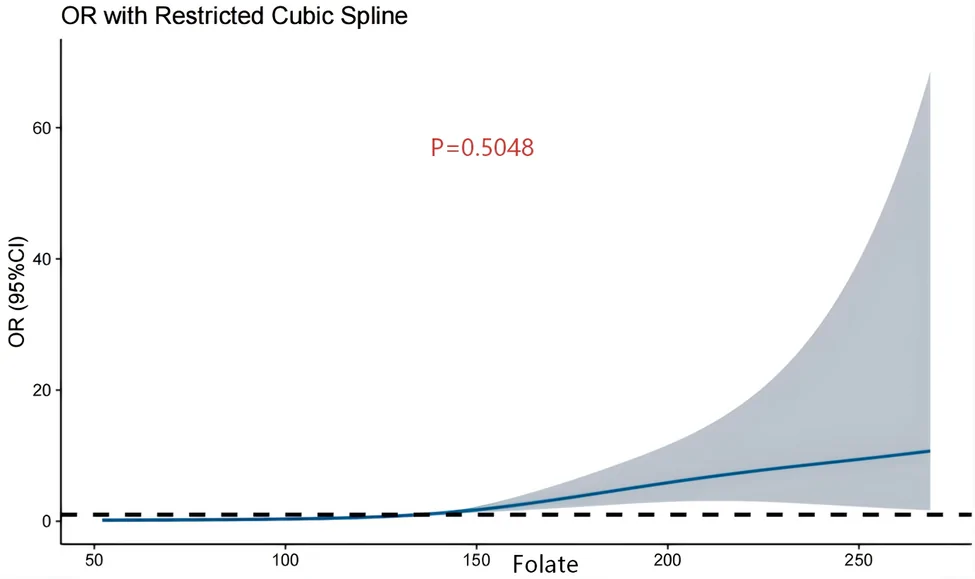
RCS analysis of folate intake and the risk of MDD in adolescentsRCS, Restricted Cubic Spline

The RCS analysis was used to assess potential nonlinear associations between dietary nutrient intake and the risk of MDD. PUFA (*P* for non-linearity = 0.0057), anthocyanins (*P* for non-linearity = 0.0068), vitamin B12 (*P* for non-linearity = 0.004), and alcohol intake (*P* for non-linearity = 0.0120) showed significant nonlinear association with MDD risk. In contrast, selenium (*P* for non-linearity = 0.1423), zinc (*P* for non-linearity = 0.0928), folate (*P* for non-linearity = 0.5048), carbohydrates (*P* for non-linearity = 0.5243), and dietary fiber (*P* for non-linearity = 0.1585) showed linear association with MDD risk in adolescents (Figs. 1, 2, 3, 4, 5, 6, 7, 8 and 9).

## Discussion

The present study demonstrated that a higher E-DII score was significantly associated with an increased risk of MDD in adolescents, indicating that a more pro-inflammatory dietary pattern may contribute to depressive vulnerability during early life. This finding is generally consistent with international studies reporting positive associations between dietary inflammatory potential and depression in adolescents and adults from Australia, Iran, the United States, and Europe [15–18]. Importantly, our study extends previous evidence to adolescents in northern China, where traditional wheat-based dietary habits differ substantially from those reported in other regions. These findings provide novel regional evidence that dietary inflammatory potential may be relevant to adolescent depression across different cultural dietary backgrounds.

Previous studies have not always reported consistent associations between dietary inflammation and depression. Several factors may explain these discrepancies. First, study populations differed substantially, with some investigations focusing only on female adolescents while others included mixed adult populations [19–21]. Second, dietary structure varies across geographic regions and may alter the inflammatory effects of specific nutrients [22]. Third, differences in study design, sample size, depression assessment tools, and covariate adjustment strategies may have contributed to heterogeneity between studies. Finally, sex-related hormonal differences may partly modify the relationship between dietary inflammation and depressive symptoms [18, 23].

In addition to the overall inflammatory dietary pattern, several individual nutrients were associated with depression risk. Higher carbohydrate intake was positively associated with MDD risk, which is consistent with previous studies linking excessive refined carbohydrate intake to metabolic dysregulation, systemic inflammation, and depressive symptoms [24–26]. Alcohol intake was also positively associated with adolescent depression in our study. Although some adult studies have reported potential protective effects of very low alcohol intake [27], such findings should not be extrapolated to younger populations because adolescence represents a critical period of neurodevelopment. Alcohol may disrupt emotional regulation, sleep quality, and brain maturation, thereby increasing susceptibility to depression. From a public health perspective, complete abstinence from alcohol should be strongly encouraged during adolescence [28].

A notable finding was the positive association between selenium intake and MDD risk. Selenium is essential for antioxidant defense and neuronal function, but accumulating evidence suggests that its relationship with mental health may follow an “optimal range” pattern rather than a simple linear association. Both selenium deficiency and excessive exposure may adversely affect mood regulation [29–31]. In our study, selenium intake was substantially higher among adolescents with MDD, suggesting that excessive intake may disturb redox balance or neuroendocrine homeostasis. This observation supports the hypothesis that maintaining selenium within an appropriate physiological range may be more important than increasing intake alone.

By contrast, higher intakes of vitamin B12, folate, dietary fiber, zinc, PUFA, and anthocyanins were associated with lower depression risk, suggesting that multiple nutrients may collectively promote emotional resilience. Vitamin B12 and folate are central to one-carbon metabolism and monoamine neurotransmitter synthesis, and inadequate intake may impair neuronal signaling [32–35]. Dietary fiber may exert protective effects through modulation of gut microbiota composition and short-chain fatty acid production, thereby reducing systemic inflammation along the gut–brain axis [36, 37]. Zinc and PUFAs may further protect against depression by regulating neuroinflammation, membrane stability, and synaptic plasticity [38]. Anthocyanins possess antioxidant and anti-inflammatory properties that may reduce oxidative stress and support neuronal function [19, 39]. Together, these findings suggest that nutrient-rich anti-inflammatory dietary patterns may be more important than single nutrient supplementation for depression prevention.

Among adolescents with MDD, lower iron intake was associated with greater symptom severity. This finding is consistent with previous evidence suggesting that iron deficiency may worsen depressive symptoms through impaired neurotransmitter synthesis, hippocampal dysfunction, and dysregulation of the hypothalamic-pituitary-adrenal axis [40, 41]. Although the E-DII score tended to increase with depression severity, this association was not statistically significant, indicating that dietary inflammatory potential may be more strongly related to disease occurrence than to symptom progression after onset.

The biological mechanisms linking diet and adolescent depression are likely multifactorial. Pro-inflammatory diets may increase circulating cytokines, alter intestinal permeability, and disrupt gut microbial composition, thereby influencing central nervous system signaling through immune, endocrine, and neural pathways [42]. At the same time, inadequate intake of neuroprotective nutrients may impair neurotransmitter synthesis and reduce BDNF expression, compromising synaptic plasticity and emotional regulation. These interconnected pathways may explain the observed associations between dietary factors and adolescent depression.

## Conclusion

The relationship between dietary intake and health outcomes is complex and interconnected, involving multiple biological pathways. This study employed the E-DII to explore the potential correlation between dietary nutrient intake and adolescent MDD, accounting for various confounding factors. Participants were recruited using gender- and age-matched methods, and relevant variables were adjusted in the analysis to reduce bias. Data collection covered major components of the Chinese diet, with trained professionals ensuring objectivity and accuracy.

Study underscores a positive correlation between the E-DII and both the risk and severity of adolescent depression, emphasizing the critical role of dietary factors in the prevention and management of adolescent depression. However, several limitations should be noted. Firstly, the case-control design precludes any inference of causality. Secondly, assessing dietary nutrient intake rather than in-body markers may result in collinearity issues, as many nutrients coexist in the same food sources and cannot be independently linked to MDD risk. Although several demographic and additional clinical variables were adjusted for in the present analysis, additional factors such as blood biochemical markers, family environment, personality characteristics, and socioeconomic status were not collected and may have contributed to residual confounding. Future research should focus on longitudinal studies that evaluate dosage levels of nutrient intake and their impact on disease development.

## Supplementary Information

Below is the link to the electronic supplementary material.

Supplementary Material 1

## Data Availability

The data that support the findings of this study are available on request from the corresponding author upon reasonable request.

## Abbreviations

DII: Dietary Inflammatory Index
E-DII: Energy-adjusted Dietary Inflammatory Index
MDD: Major Depressive Disorder
FFQ: Food frequency questionnaire
