# Supplementary material for: The relationship between dietary nutrient intake and adolescent depression based on the dietary inflammation index: a case-control study
Source: BMC Psychiatry. 2026 Aug 3;26:848. doi: 10.1186/s12888-026-08425-y (PMC13721568; doi:10.1186/s12888-026-08425-y)
Supplement: Supplementary file 1 [file 12888_2026_8425_MOESM1_ESM.pdf]

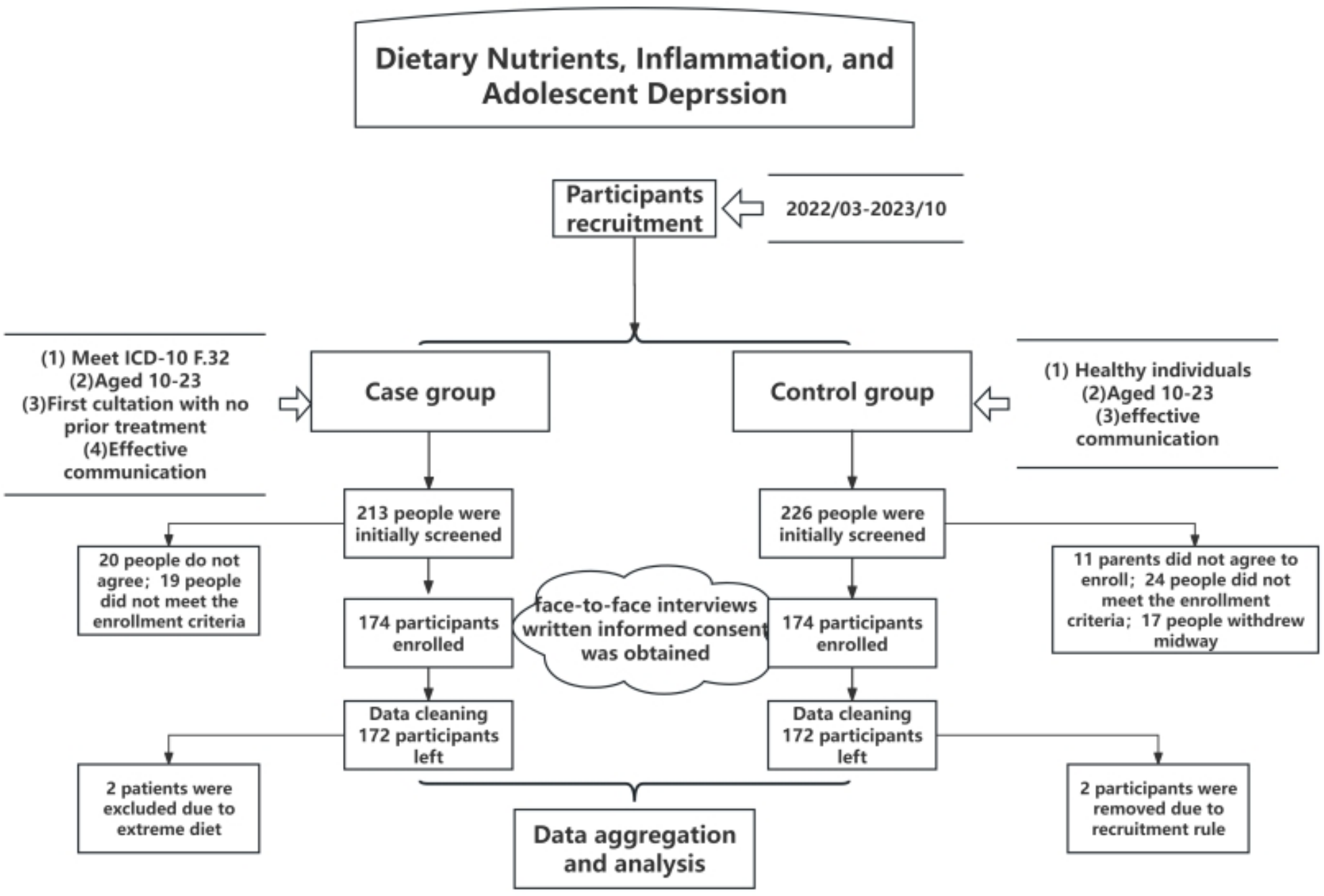

Supplementary Figure. Participants recruitment flow chart
